# Quarterly Retrospect

**Published:** 1862-01

**Authors:** 


### *Quarterly Retrospect.*

*January*, 1862.

"A SAD tale's best for winter," says Mamillius in the "Winter's Tale." The Quarter's Retrospect will, in some sense, prove a gloss upon this line of the poet, for it will consist of a bare record of murder and suicide; and if the sad recitals we are about to note, should induce greater attention on the part of our readers in the winter time, than at any other period of the year, to the circumstances out of which they have arisen, then we may admit that a sad tale is best for winter, in a better and higher sense even than the poet meant.

Few circumstances have ever excited greater horror than the series of military murders which have been perpetrated in this country within the past twelve months. The petty motives which have led to the perpetration of these horrible deeds, the cool deliberation and determination with which they have been carried into effect, and the utter indifference of the murderer to, nay, the absence of all wish to escape from, the consequences of his foul act, are almost without parallel in the history of modern crime. In connexion with these murders we would direct attention to a fact which in all probability has a close and important relation to the circumstances which have prompted such deadly acts of revenge.

From the Statistical Report of the Health of the Army in 1859, recently published by the Army Medical Department, we learn that, in that year, 20 ascertained suicides occurred among a strength of 71,715 men in the United Kingdom. The 19th report of the Registrar-General shows that, in the five years 1852-56, the proportion of suicides in England, among *males*, in 1,000,000 living at all ages, was 85·1; and the proportion among the same number living at the *military* age (20 to 40), may be estimated approximatively at 124·6; the proportions in decennial periods from 15 to 55 years of age being as follows:—

| | | | |
|---|---|---|---|
| 1 to 25 | " 40·1 | 35 to 45 | " 138·4 |
| 25 " 35 | " 30·0 | 45 " 55 | " 240·0 |

If, now, we reduce the army returns of suicide for 1859 to the same ratio, we find that they give a proportion of not less than 278·8 in 1,000,000 living at the military age; that is to say, a proportion *more than double that found in civil life!*

We cannot help thinking that the causes which lead to this extraordinary excess of suicide among the troops on the Home Stations, and those which have given rise to the recent outbreak of murders, have much in common. It is surmised that the murders were in no small degree dependent upon a feeling of irritation, which is believed to be too common among the soldiers, and which is provoked by vexatious obstacles being thrown in the way of their making known their just complaints. However this may be, there can be no question that the army returns of suicide confirm the necessity, made but too apparent by the recent murders, for a careful inquiry into such grievances as may exist among the troops.

The series of military murders, unfortunately, do not stand alone as recent exceptional instances of crime of the deadliest character. In our last Retrospect we were compelled to omit the notice of one or two remarkable examples which had occurred in the previous quarter; but these may fittingly find a place here.

At the Summer Assizes for Chester two children, each aged only *eight years*, were placed upon their trial on a charge of wilful murder. From the evidence it appeared that they had got hold of a child two years and a half old, had taken him into a field, stripped him naked, beaten him so dreadfully that his body was one mass of bruises; then dragged him to a pool close at hand, put clogs upon his feet that he might sink the quicker, and, finally, pushed him into the water and there left him drowning!

In the past quarter a youth aged eighteen years, was tried at the Old Bailey for murdering his sister, aged between ten and eleven years, under the following circumstances. They had quarrelled (not an unusual circumstance, it appears), when he deliberately strangled her with a piece of fine cord, and cast the body into a coal-cellar. Some little time afterwards, when his sister had been missed, he told one of his comrades what he had done, saying, "It's no use looking after my sister Polly; go back, and tell my father if he wants my sister Polly, he will find her in the coal-cellar, with a rope round her neck strangled." To the policeman, who arrested him for the murder, he remarked: "I did it; she aggravated me to it." He showed no emotion at the time of his arrest, or in the course of the trial; not even when sentence of death was passed upon him. The sentence has since been commuted (solely, it would seem, on account of the youth of the culprit) to imprisonment for life.

A remarkable example of murder and attempted suicide by a person much advanced in life, was tried at Mold, in the North Wales Circuit, on the 2nd of August last. The prisoner, a female, aged seventy-seven years, was arraigned for the murder of her husband, aged eighty years. The crime was perpetrated on the 22nd of May last. The prisoner and her husband had always lived on good terms, but had latterly been in indigent circumstances, and received parochial relief, and he had been an invalid about four months, and at the time of his murder was daily expected to die. Between six and seven o'clock in the morning she cut her husband's throat with fatal effect, and subsequently attempted to cut her own throat, inflicting a severe wound. The only explanation given by the prisoner of her motive for committing the crime was that "having seen better days, and now being poor, and Thomas ill, calling every hour for attendance, she preferred dying to living." It was stated by a niece in evidence that the husband had said to her that her aunt "was rather strange;" and Mr. Peter Parry, coroner for the county of Flint, and a medical practitioner of old standing, stated that when he went to view the deceased, he saw the prisoner, and that her "eyes were very penetrating and wild." He was of opinion that she was insane. Other evidence, not reported, was also given in support of the plea of insanity. The jury returned a verdict of *guilty*, but recommended the prisoner to mercy on account of her great age. The verdict created considerable murmuring in the court. Sentence of death was passed, but, we presume, it has not, nor will not (supposing that the woman is still alive), be carried into effect.

The whole case is of singular interest, and it is to be regretted that we have not a fuller report than that from which we have derived the foregoing meagre details. The mental state of the woman merited a much more close and systematic study than that to which it appears to have been submitted. It would be interesting to learn what her state has been since sentence of death was passed upon her.

As a pendant to these cases, the following story of atrocious villany, told by the Italian correspondent of the *Times* (Nov. 29th), in a letter dated Turin, Nov. 25th, is worthy of quotation:—

"As a mere psychological curiosity, and an evidence of hardened villany which it could hardly have been deemed possible for human nature to attain, I beg to be allowed to translate a few extracts from the reported trial of that Antonia Boggia, charged with a multiplicity of deliberate murders at Milan, to whom I have repeatedly alluded in some of my foregoing letters. You are aware that this Boggia was a house-porter, in easy circumstances for one of his standing in life, very assiduous in the discharge of his religious duties, somewhat over-demonstrative in his display of zeal and devotion, and a darling of the Milan priesthood, who once got him out of a sanguinary scrape, for which, but for their interference, he would have been, under the Austrian Government, hanged in time to disable him from the perpetration of subsequent offences. Imagine, says the report,

"A little man about 65 years of age, with venerable grey hair carefully smoothed down on the temples and back of the head, an easy cheerfulness of countenance, an imperturbable calmness of speech, a spotless white neckcloth; the whole outward man would lead you to look upon him as a poor aged wight brought into difficulty by some mistake, or in consequence of some deep-laid scheme of calumny. The President asks him by what circumstances he was led to do away with his last victim, the woman Perocchio, 66 years old, who had welcomed him to her house with the most perfect trust. Boggia begins by rubbing his hand, takes his handkerchief out of his pocket, wipes his mouth; then pulls out his snuff-box, takes a good pinch; then, without a wink of the eye, no faltering of the voice, without a glimpse of remorse or compunction, he thus tells his atrocious tale:—

"What can I say to you, my Lord President? We were there, all alone; the old woman smiled: a whim or inspiration came upon me; I took up my hatchet, and let it go at her head with so good an aim that she did not utter one cry; she was knocked down instantly, and died quite easy. When she lay on the ground, stretched out, I sat down for a quarter of an hour looking at her, and as I looked a fit of laughter seized me. I then went out for a little air, and came back to sleep. On the morrow I cut off the woman's legs, to be able to put her in my basket (a kind of large basket with handles, used by street porters in Italy to carry burdens on their shoulders), to make it one job only as I carried her to my cellar. When I had her in my cellar I dug a goodly grave (*una brava fossa*) along the wall, took out the pieces of the old woman, laid them down in the grave very nicely, stretched out at full length, and there was an end of it.

"And Ribbone? What of Ribbone? asked the Court. This Ribbone was an old friend of Boggia; he lived in the same house,—a good man, fond of Boggia's children, who patted them on the head, bought them penny toys, took them out for a walk, and was quite intimate with the family. Boggia asked his friend for a loan of twenty lire. Ribbone promised to try to get them, but Boggia's impatience got the better of him. He found some pretext to decoy his poor friend into his cellar, asked him to look for something he had dropped on the ground, and as the other stooped he was over him with the formidable hatchet which he had secreted under his cloak, and with one stroke on the nape of the neck he levelled him, stone dead, with the ground.

"But why did you kill him?

"Simply because he did not procure me the twenty lire I wanted.

"On another occasion, after killing one Mazza, 'he went out of his cellar for a little air,' as he said, 'and walked along the canals to see the boats loading; then came back at night, and dug the usual grave. But the grave was not long enough. He doubled up the corpse as he best could, and left it to keep company with the other victim."

We have again to record several instances of *murder by lunatics.* The following case, tried at the Circuit Court of Glasgow, on the 27th September last, is of considerable interest, the prisoner being without hesitation acquitted on the ground of insanity, it being shown that he was suffering from a peculiar form of nervous excitement, accompanied with mental perversion, induced by intemperance, at the time when the crime of which he was charged was committed.

On the 4th of August last, Robert Pattison, at one time an assistant-inspector of the poor in Glasgow, murdered his daughter, Magdalene Pattison, aged 19 months, by cutting her throat. The evidence adduced at the trial, bearing upon the mental state of the prisoner at the time of the murder, and which led to a verdict of not guilty on the ground of insanity, was chiefly as follows:—

"Margaret Crawford or Whiteside—I reside in Meuse Lane, Cowcaddens. I recollect of panel coming to my house between half-past 7 and 8 on Sunday night. I was struck by his appearance. He did not look as he used to do. He was raised-like, and looked as if something had been troubling him. He looked as if he had been drinking. He had neither collar or necktie. He had a cap on his head. I never saw him with a cap on before. He was always in the habit of wearing a hat. I clapped him on the breast, and said, 'Bob, you have not been at home all night;' and he said he had. He asked me for something to put round his neck, and I went to get a napkin for him. I went to another room, and when I came back I found him crying, with his hands before his face. He told me he was going to the Police Office, and on leaving he shook hands with me, and said I would never see him again. I asked him how the children were, and he said, 'Is it hers or mine?' I said, both, and he replied they are well; mine are outside, meaning they were boarded out. I gave him a necktie, and tied it on his neck myself. He was about five or ten minutes in my house. When he said he was going to the office, I thought he had been in some of 'thae hooses,' and got himself stripped, and I advised him to go home, and not go to the office. He said he would not go home, but there was a boat leaving Clyde that night, and he would be off. He had nothing in my house but a drink of cold water. That was all he asked for. He did not talk sensibly; he was wavering back and forward. The man was raised-like, but I understood all he said. I formed the opinion that he was not sensible from his having come into my house on a Sunday without a collar or necktie. He seemed to be frightened-like when he came in. His hands were shaking. My daughter was at door when he came up and asked if this was Whiteside's, and she said it was. He then said it was all right, and walked into the kitchen.

"By Mr. Miller—I knew Pattison quite well. He could not but know my house, and I was rather surprised that he asked my daughter that question. He was always kind to his children. When he asked a drink of water, I asked him if he would take anything in it, but he declined. It was daylight when he came to my house. His appearance altogether was very noticeable. When he left, I cannot say he staggered; he held his head down. I was under the impression at the time that all was not right, and I could not sleep all night in consequence. I was afraid that something serious had happened.

"Henry Douglas, turnkey in the Northern Police Office—I was on duty on the evening of Sunday, 4th of August. I recollect of Pattison coming to the office that night about ten minutes to 8. He had on a cap and an old tie, and was otherwise dressed. He asked if Mr. Bowker was in; I said he was not in, as he was on leave. He asked who was acting for him, and he was told it was constable Ellis. He said, 'How am I to know Ellis?' and I said he should come back when he was sober. He smelt strongly of drink, and appeared to be the worse of it. He then opened the bar door and said, 'I give myself up; I have done a murder.' I said, 'Get out of this, sir;' and then turned round to the sergeant, who was acting-lieutenant, and said, 'This man's in the blues, I think.' We then caught hold of him to put him outside. He insisted upon getting in, but we put him out, when Mr. Nelson came in and told us to lock him up for protection, as he was under the influence of liquor. He was searched and locked up. I left that evening at 9 o'clock, came on duty next morning, and found that panel had been detained on a charge of murder. After he had been brought before the magistrate, I again locked him up. I asked him if he wished any breakfast, and he said he would rather have half a glass of whisky, if I would ask Mr. Taylor. He got the spirits. I said this was a bad job for him, and he said he was done for now. On Sunday he spoke like a man in his senses, but his eyes were rolling in his head. Did not observe him shaking. He insisted, when we put him out, to be in again. He did not appear to be frightened. He walked quietly from the bar to the cell. He shaked very much on the Monday morning, as if after hard drinking, but he was composed and speaking quite intelligibly. He did not seem in terror. He came on Friday night in the same state as he was in on the Sunday, and asked for Mr. Bowker. When he got the same answer, that Mr. Bowker had gone on leave, he said, 'You are a pair of d——d impertinent fellows.'

"John H. Nelson, Assistant-Superintendent, Northern District, Glasgow Police, examined by Mr. Thomson—Remembers the prisoner being given into custody on the evening of 4th August, near 8 o'clock. Prisoner was the worse of liquor, and was locked up. When he went home he was apprised that a child had been murdered. He went to the house, and caused the body to be removed to the Northern Office. He then got prisoner out of his cell, and he told him that he had killed his child. Prisoner said, 'Is it possible?' Witness said, 'Yes.' He asked which of the children it was, and witness said it was his youngest child, Magdalene. Prisoner said, 'That canna be; it was the child I loved the most.' Prisoner then burst into tears, seized hold of the lieutenant's bar, and said he was like to faint. He got a little water and recovered. He then said he was done for in this world. He was nervous and shaking, as he was at 8 o'clock, when witness first saw him, but he appeared to be much more agitated. His conduct impressed me that he was a man considerably addicted to drink, and to be then labouring under the effects of it. He was not exactly in a delirious state, or in the 'blues,' but very nervous. He was asked if he had any razor, and he said it was on the dresser in a case. Found spots of blood on the breast of his shirt. Prisoner said he was not aware that there was any blood there, and that he did not know how it came there. (Identified shirt, also razor.)

"Cross-examined by Mr. Miller.—He ordered prisoner's braces and neck-cloth to be taken away, in case he might commit suicide. On the Monday morning he seemed more composed, but unconscious of what had taken place. He appeared to have forgotten what witness had told him on the preceding evening. When he first saw prisoner at eight o'clock that Sunday evening it appeared that his mind was a little wandering. His impression was that it arose from habitual drinking.

"Daniel Taylor, Lieutenant, Northern District, corroborated testimony of preceding witness. Prisoner said to Mr. Nelson the Sunday evening after the murder, 'Surely there is something far wrong when you are examining me so closely.' Witness was under the impression that the prisoner then did not know what had taken place—not that he was assuming ignorance. Prisoner said if he got a little spirits he might get a sleep, which might enable him to remember what Mr. Nelson had charged him with. He got a little spirits about three o'clock in the morning. About an hour after witness saw him, prisoner said he had had a strange sort of dream, that he was afraid he had done something to that child with a razor. Afterwards he said, 'It's a terrible job; I am afraid I'm done for in this world.' He seemed very much distressed. Witness formed no impression on that Sunday night and Monday morning as to prisoner's state of mind than he would as to any other drunk man. He showed no symptoms of being in the 'horrors.'

"Dr. Miller examined.—I drew up a report after examining the body of Magdalene Pattison. [Witness here read the report, which stated that death had been caused by excessive hemorrhage from a wound in the neck. A razor might have inflicted the wound. Witness also read two reports, drawn up by himself and Dr. Stewart, regarding the death of the child, and the condition of the prisoner. The latter report stated that the general appearance of the prisoner was that of an habitual drunkard, but he was perfectly sane on the 5th of August.]

"Examination resumed—The prisoner entered pretty fully into his history for some time past. He said that he had drunk heavily for some time, and more so since the death of his wife. I saw the prisoner at one o'clock on

Monday morning, the 5th of August, in the Northern Police Office. He was in a very confused, perplexed state, and there were symptoms which pointed to temporary insanity. He was very weak-looking, something like a person recovering from a debauch. He was nervous, and had a scared look. His pulse was quick, and his tongue furred. His eyes were rolling a little. From the first story I heard, it increased my impression that the prisoner was temporarily insane at the time of committing the murder. I am now quite satisfied that the crime was committed while he was in a state of temporary insanity.

"Dr. Stewart was next examined, and spoke to the reports which had been drawn up by Dr. Miller and himself. He continued—I have heard the evidence to-day, and the impression that I formed at first is now confirmed. It is my impression that when the prisoner committed the crime he was insane.

"This concluded the evidence for the prosecution, and Mr. Miller stated that he had medical evidence to lead; but after the evidence which had been given by the medical gentlemen for the prosecution, he did not think it necessary to lead any further evidence.

"Mr. Moncrieff then briefly addressed the jury, and said, that after the testimony of the medical gentlemen, he had no alternative but to ask for a verdict of not guilty, in respect the panel was insane when the crime was committed.

"Mr. Miller then addressed a few words to the jury, after which Lord Ivery directed them to return a verdict of not guilty.

"The jury then returned a verdict, finding the prisoner not guilty, in respect that he was insane at the time the crime was committed.

"The Court then adjudged the prisoner to be kept in close custody in the prison of Glasgow during her Majesty's pleasure."

2. The *Halifax Courier*, of November 9th, reports a case of child-murder and suicide (the throat being cut in both instances) by the wife of a farmer in comfortable circumstances, at Mythalmroyd, in the township of Wadsworth. The following summary of the circumstances attending the case is given by the reporter:—

From the evidence, it seems that Mrs. Greenwood has ever had an affection for her children which amounted almost to a disease. By two accidents which have happened to them, she has been so deeply moved that her mind has been slightly affected. The first of these was to a boy, whose life was scarcely saved from a flood; and the second and the one which really led to the deeds of blood of which we are writing, was to the deceased child Grace, who it seems, incautiously approaching a newly-calved cow, was severely injured by her; it is said trodden upon. These accidents shocked the nervous system of the too sensitive mother. Her grief and anxiety were increased when, after the lapse of a few months, the girl became at first slightly deformed, then affected mentally—losing her memory and so forth; and then to suffer from fits. Mrs. Greenwood is described as having been cheerful and high-spirited usually, but if by any turn of thought or by an expression of others, or the occurrence of any circumstance, she was led to speak of the girl, her countenance saddened, and she expressed mournfully her fears for the future of the child. Seven months ago she was confined of a baby, but failed to recover strength, as was usual with her. The fact was, her previous sufferings had begun silently to tell on both mind and body. Eventually Dr. Howard, of Hebden Bridge, was sent for, and he at once saw what was her ailment and accurately surmised the original cause—severe nervous shocks. Her appetite had gone, and while partaking of no food she was suckling a strong child. To supply milk, she drank largely of fluids, and, taking no solid food, her blood became watery, almost useless; and the doctor plainly informed the family that the child must be removed or the mother would die or go mad. Whether the child was removed does not appear, but the mother said that she had suffered much in mind and body, and that the doctor's order should be attended to. Nothing to excite special notice seems to have occurred in the interval, except the poor woman's morbid uneasiness about her poor girl's state. This uneasiness preyed on her weakened system, and the "method of her madness" in the end was to all human appearance a desire to send the poor child out of all danger of future sorrow, by cutting the thread of its existence; and herself to join it in another world by the same short and bloody path. The carefulness of the crazed was manifested in her choice of a time for her work,—when her husband was away, and having on a pretence of kindness sent the rest of the family out of the way.

3. The daily papers of November 22nd record the particulars of the murder, in Dublin, of two children, one two years old, the other three, by their father. The family were in a state of frightful destitution, and the father had been ill several days before he committed the crime. He was subsequently acquitted of the charge of wilful murder on the ground of insanity.

4. John Atkins was tried at Maidstone, December 4th, for the murder of his wife. In evidence it was shown that he had delusions, particularly as to the continency of his wife, and that he was subject to hallucinations, both of sight and hearing. A verdict of *Not Guilty* was returned on the ground of insanity.

5. The following case of *child-murder* was recorded in the *Times* of December 7th:—

An event of a most heartrending character took place on Thursday at the little village of Slaugham, situated at a distance of about six miles from Hayward's-heath. It would appear that for some time past a Mr. Agate has occupied a farmhouse in the village, and that there resided with him, among other persons, his wife and two little boys, aged respectively four and six. From the general demeanour and behaviour of the family the whole of its members were much respected. Mrs. Agate has for some short period been indisposed. No serious results were feared, however, until within the past few days, when her conduct became such as to cause her friends considerable uneasiness. In consequence she was pretty carefully watched by those around her. Things continued to proceed in this manner until yesterday morning, when shrieks were heard to proceed from the farmhouse. An alarm having been given, several persons at once went to the spot, and after some slight difficulty an entrance was effected, when the greatest consternation was caused by the scene which presented itself. The body of the younger of the boys above referred to was found in a bedroom lying in a pool of blood, which was still flowing copiously from a frightful gash in the throat. The other boy, the elder of the two, lay stretched in a state of insensibility in the same room, also suffering from wounds about the neck and throat, which bled profusely, and on a further search Mrs. Agate was discovered with wounds in her throat. Neither she nor the boy last mentioned was dead. That the unfortunate woman, in a moment of frenzy, made the onslaught on her two children and then attempted her own life is not doubted for a moment by those who now have charge of the case, every circumstance that comes to light, including the mental disease of Mrs. Agate, tending to corroborate that conclusion. The injured woman and child remain at the farmhouse under medical care.

